# Soft tissue tenodesis of the long head of the biceps tendon associated to the Roman Bridge repair

**DOI:** 10.1186/1471-2474-9-78

**Published:** 2008-06-04

**Authors:** Francesco Franceschi, Umile Giuseppe Longo, Laura Ruzzini, Giacomo Rizzello, Nicola Maffulli, Vincenzo Denaro

**Affiliations:** 1 Department of Orthopaedic and Trauma Surgery, Campus Biomedico University, Via Alvaro del Portillo, 200, 00128 Trigoria, Rome, Italy; 2 Department of Trauma and Orthopaedic Surgery, University Hospital of North Staffordshire, Keele University School of Medicine, Stoke on Trent, ST4 7LN, UK

## Abstract

**Background:**

Rotator cuff tears are frequently associated with pathologies of the long head of the biceps tendon (LHBT). Tenotomy and tenodesis of the LHBT are commonly used to manage disorders of the LHBT.

**Methods:**

We present an arthroscopic soft tissue LHBT tenodesis associated with a Roman Bridge (double pulley – suture bridges) repair

**Results:**

Two medial row 5.5-mm Bio-Corkscrew suture anchors (Arthrex, Naples, FL), double-loaded with No. 2 FiberWire sutures (Arthrex, Naples, FL), are placed in the medial aspect of the footprint. A shuttle is passed through an anterior point of the rotator cuff and through the LHBT by means of a Penetrator or a BirdBeak suture passer (Arthrex, Naples, FL). A tenotomy of the LHBT is performed. All the sutures from the anteromedial anchor are passed through a single anterior point in the rotator cuff using a shuttle technique. All the sutures from the posteromedial anchor are passed through a single posterior point in the rotator cuff. The sutures in the medial row are tied using the double pulley technique. A suture limb is retrieved from each of the medial anchors and manually tied as a six-throw surgeon's knot over a metal rod. The two free suture limbs are pulled to transport the knot over the top of the tendon bridge. The two free suture limbs are then used to produce suture bridges over the tendon, using a Pushlock (Arthrex, Naples, FL), placed 1 cm distal to the lateral edge of the footprint. The same double pulley – suture bridges technique is repeated for the other two suture limbs from the two medial anchors.

**Conclusion:**

This technique allows to perform a double pulley – suture bridges repair for a rotator cuff tear, associated with a soft tissue tenodesis for the management of LHBT pathology. The tenodesis of the LHBT is performed just with the passage of a shuttle inside the LHBT, after passing it through the anterior portion of the rotator cuff, with successive detachment of the LHBT from the glenoid. It is a technically easy procedure which can be performed relatively quickly, and does not require additional fixation.

## Background

Rotator cuff tears are frequently associated with pathologies of the long head of the biceps tendon (LHBT) [1,2]. Tenotomy and tenodesis of the LHBT are commonly used to manage disorders of the LHBT [2]. The differences may be cosmetic, anticipated activity demands, post operative compliance. The indications for either procedure is a painful shoulder felt to be related to an abnormal biceps tendon. Tenotomy is indicated if the tear is irreparable, or if the patient is older and not willing to participate in the rehabilitation programme [2-5].

LHBT tenodesis is indicated in severe biceps tendinopathy, partial LHBT tendon tear (greater than 50% of tendon diameter), full-thickness biceps tendon tears, medial subluxation of the tendon, or nonreparable SLAP lesion [2,3,6]. LHBT tenodesis is also recommended for younger, active patients [2,3,7]. Several open and arthroscopic tenodesis techniques have been described, including suture anchors into the bicipital groove [8-10] and interference screw fixation in a reamed humeral tunnel [11]. These techniques require increased operative time for the use of a separate mean of fixation. Soft tissue tenodesis can be an alternative to them [12-14]. None of the available tenodesis techniques is superior to another in level I studies, and soft tissue tenodesis has not been demonstrated to produce inferior clinical results than other biceps tenodesis techniques.

We present an arthroscopic soft tissue LHBT tenodesis associated with a Roman Bridge (double pulley – suture bridges) repair [15].

This techniques allow to perform a double pulley – suture bridges repair for a rotator cuff tear [15] and achieve a soft tissue tenodesis for the LHBT pathology, avoiding the use of an additional fixation for the LHBT.

## Methods

All procedures described in the present article were approved by the Local Ethics Committee of the Campus Biomedico University, Rome, Italy, and all patients gave their written consent.

## Results

### Arthroscopic technique

Patients undergo brachial plexus block and are placed in a lateral decubitus position. The arm is suspended at approximately 45° of abduction and 20° of forward flexion. Distraction of the shoulder joint is accomplished with 4.5 to 6.5 kg of traction. Four to six portals are used. A posterior portal is produced, and a routine diagnostic arthroscopy is performed to evaluate the extent of the rotator cuff tear, lesions of the biceps tendon, and other associated lesions. The main subacromial portals are the postero-lateral viewing, the antero-lateral, and the lateral working portal, with an 8.25 mm cannula.

A spinal needle is introduced percutaneously to determine the precise location for placement of the antero-lateral portal produced approximately 2 to 3 cm anterior and lateral to the antero-lateral corner of the acromion. If the subscapularis tendon is involved, an anterior mid-lateral portal is produced just superior to the lateral half of the subscapularis tendon. The lateral portal is used to mobilize the rotator cuff back to its bony insertion. The mobility of the rotator cuff is assessed.

Using a burr through the lateral portal, the footprint of the greater tuberosity is abraded.

**Figure 1:**
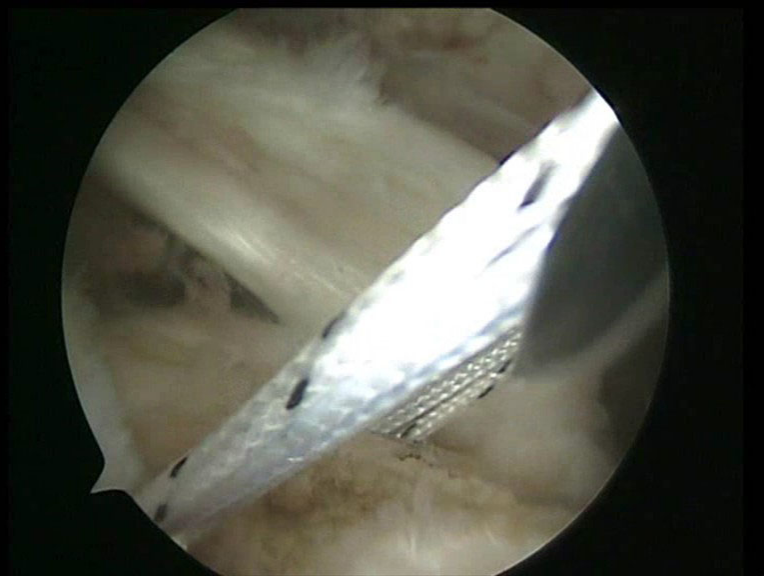
Two medial row suture Bio-Corkscrew anchors (Arthrex, Naples, FL), each double-loaded with No. 2 FiberWire sutures (Arthrex, Naples, FL), are placed in the medial aspect of the footprint, just lateral to the articular surface of the humeral.

Two medial row 5.5 mm Bio-Corkscrew suture anchors (Arthrex, Naples, FL), which are double-loaded with No. 2 FiberWire sutures (Arthrex, Naples, FL), are placed through percutaneous punctures in the medial portion of the footprint, just lateral to the articular surface of the humeral head (Fig 1). The first anchor is placed in the anteromedial aspect of the footprint. The second anchor is placed approximately 1.5 to 2 cm posterior to the first anchor.

**Figure 2:**
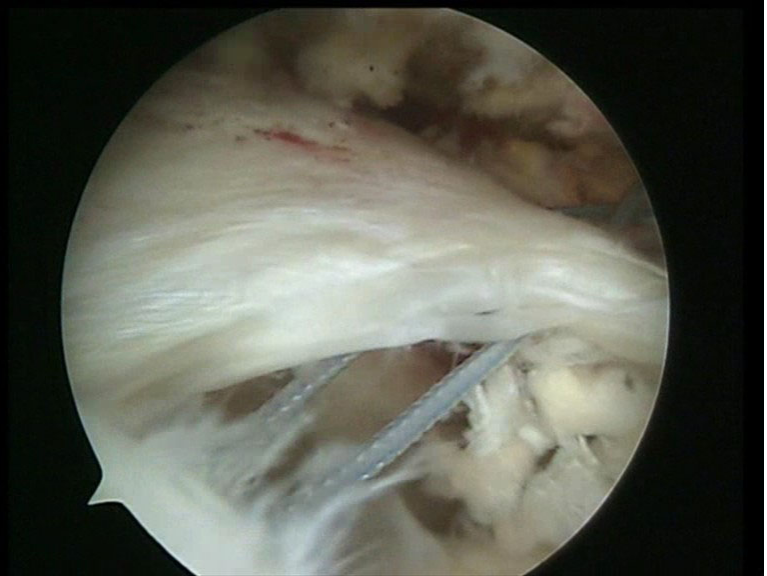
Two side to side sutures are passed through the anterior and posterior aspect of the rotator cuff.

**Figure 3:**
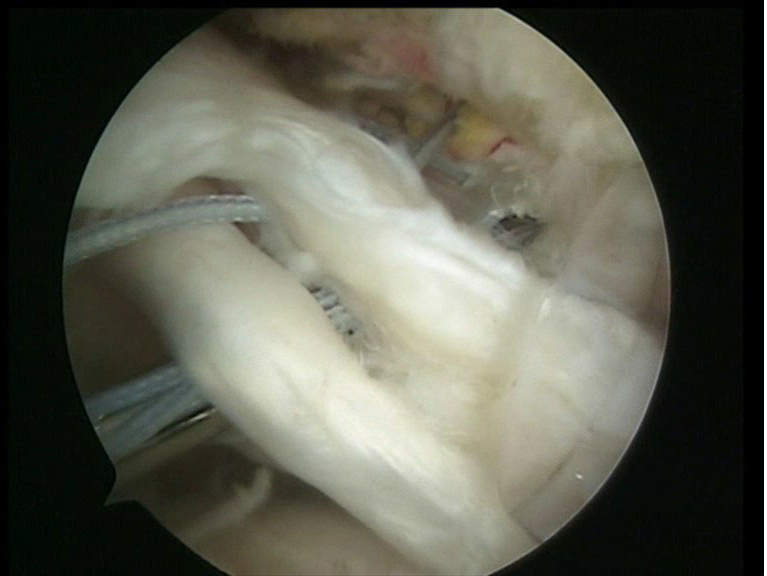
A shuttle is passed through the anterior portion of the rotator cuff and through the long head of the biceps tendon by means of a Penetrator (Arthrex, Naples, FL).

**Figure 4:**
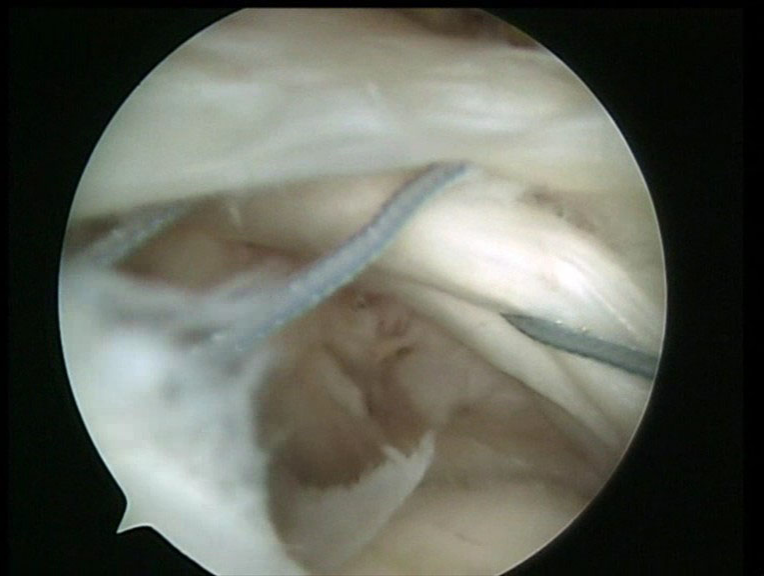
The shuttle is passed through the anterior portion of the rotator cuff and through the long head of the biceps tendon.

**Figure 5:**
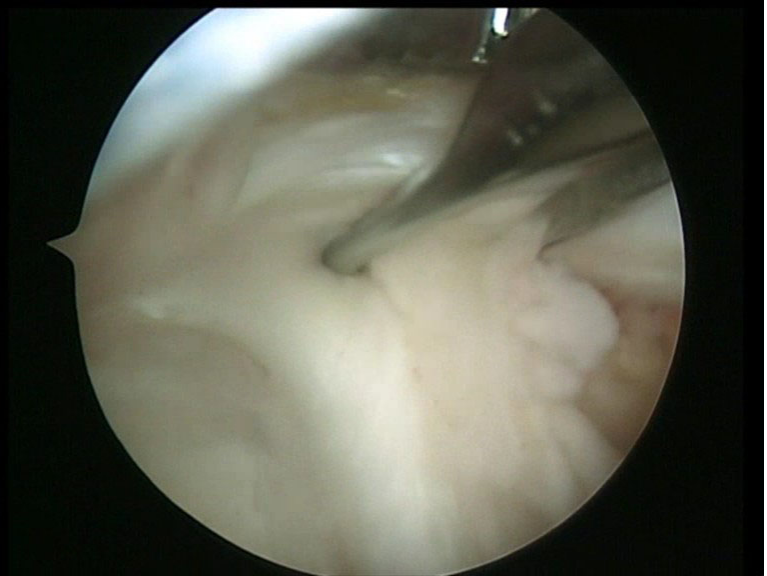
A tenotomy of the long head of the biceps tendon is performed.

**Figure 6:**
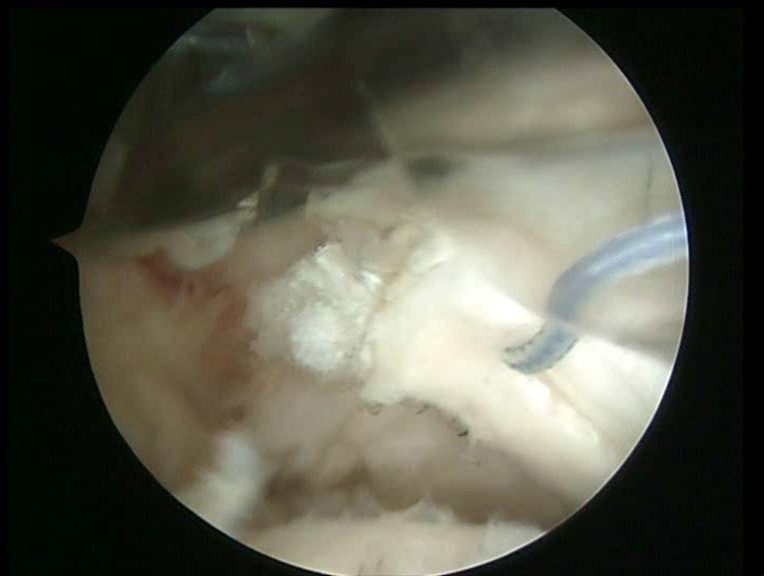
The long head of the biceps tendon is detached from its insertion to the glenoid.

**Figure 7:**
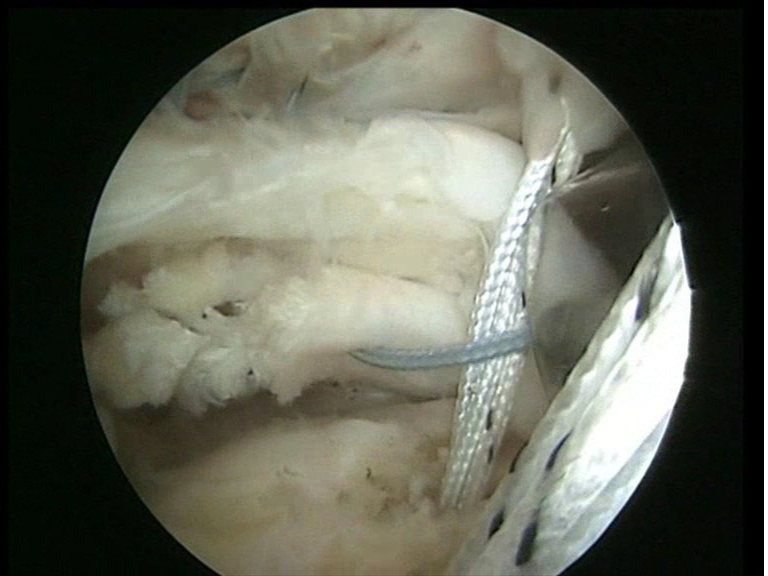
A shuttle passing through an anterior point of the rotator cuff and through the tenotomized long head of the biceps tendon.

**Figure 8:**
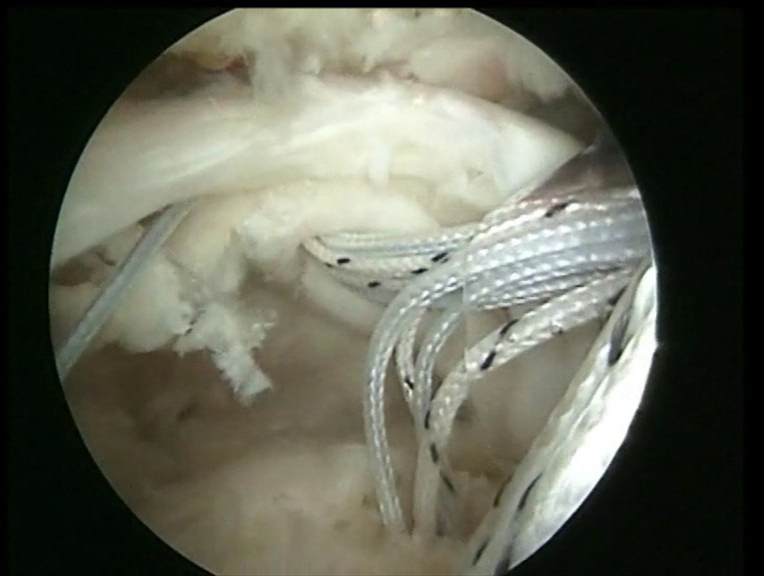
All the sutures from the anteromedial anchor are passed through a single anterior point in the rotator cuff using the shuttle.

**Figure 9:**
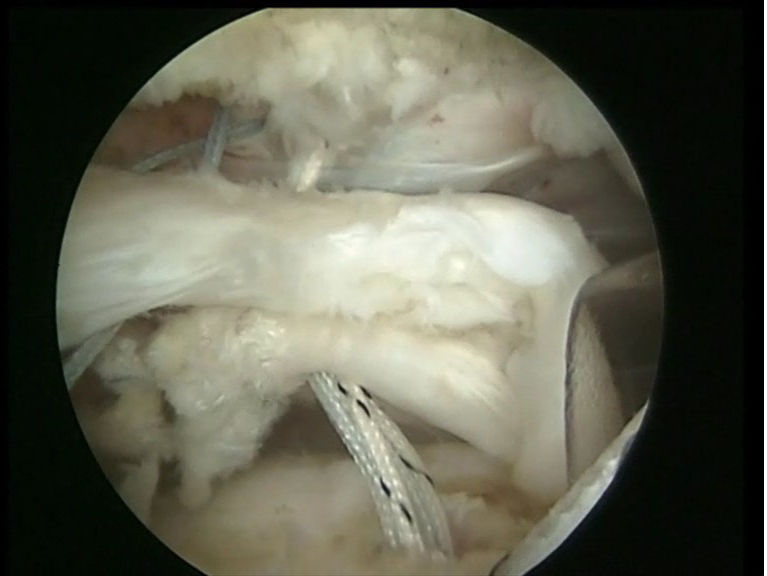
The sutures are passed through the tenotomized LHBT and through a single anterior point in the rotator cuff.

**Figure 10:**
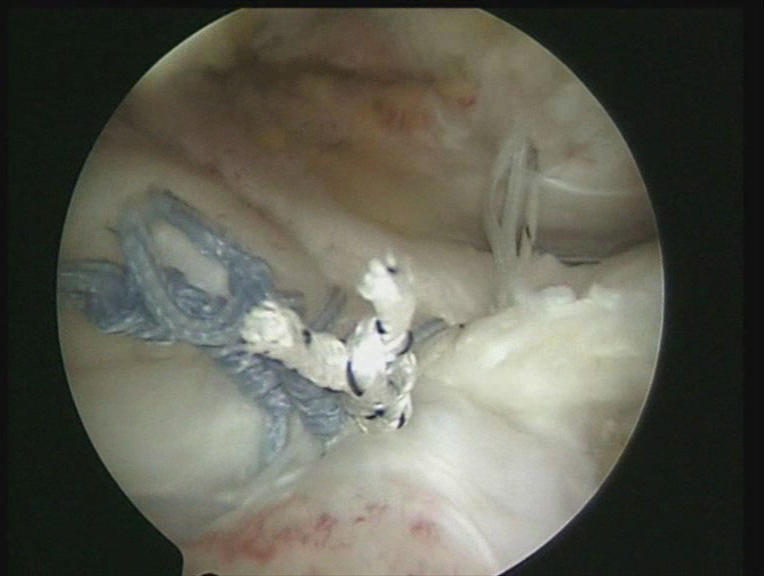
The two free suture limbs are pulled to transport the knot over the top of the tendon bridge.

**Figure 11:**
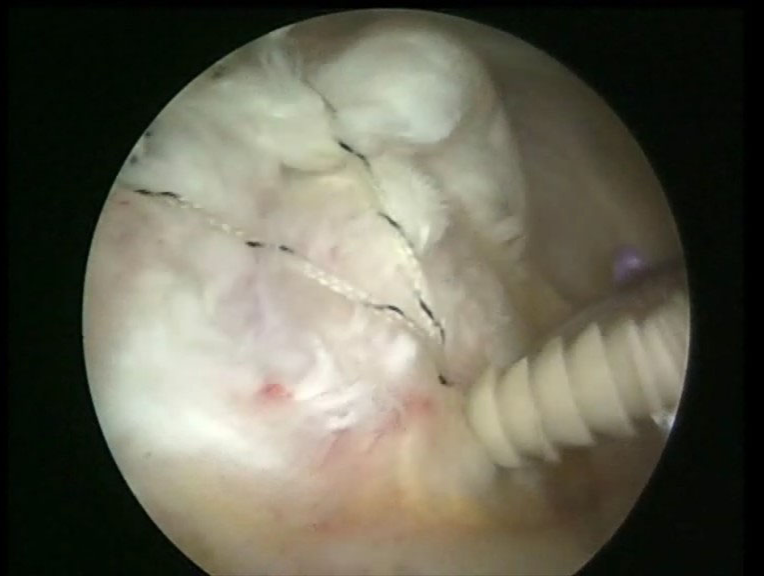
Two free suture limbs are used to produce suture bridges over the tendon, using a Pushlock (Arthrex, Naples, FL), placed 1 cm distal to the lateral edge of the footprint.

**Figure 12:**
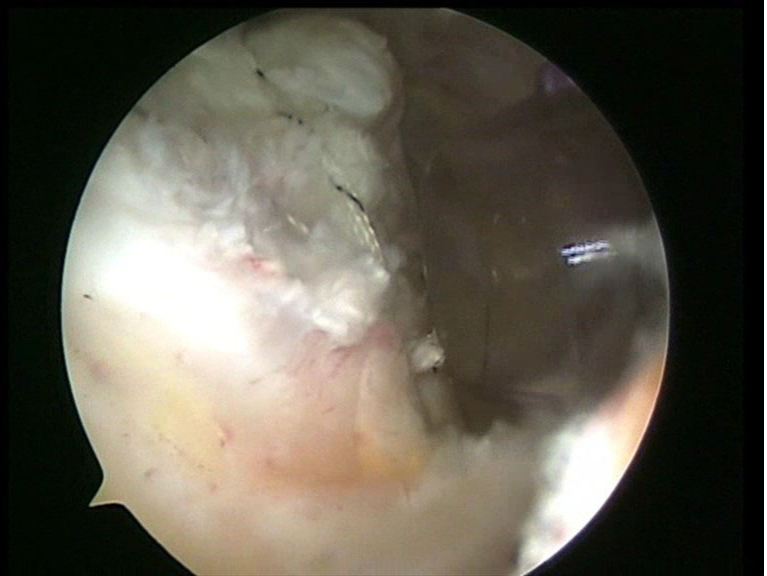
The two free suture limbs used to produce suture bridges over the tendon are cut.

One or two side to side sutures can be performed depending on the size of the lesion (Fig 2). A shuttle is passed through an anterior point of the rotator cuff and through the long head of the biceps tendon by means of a Penetrator or a BirdBeak suture passer (Arthrex, Naples, FL) (Fig 3, 4). A tenotomy of the LHBT is performed (Fig 5, 6). All the sutures from the anteromedial anchor are passed through a single anterior point in the rotator cuff using a shuttle technique (Fig 7, 8, 9). All the sutures from the posteromedial anchor are passed through a single posterior point in the rotator cuff. The side to side sutures are tied, and the sutures in the medial row are tied using the double pulley technique. A suture limb is retrieved from each of the medial anchors through the lateral portal, and manually tied as a six-throw surgeon's knot over a metal rod. A tendon grasper introduced through a lateral portal is used to grasp the medial aspect of the rotator cuff tendon, which is pulled laterally toward the bone bed. The two free suture limbs are pulled to transport the knot over the top of the tendon bridge (Fig 10). This technique is called the "double-pulley" technique, because the eyelets of two suture anchors are used as pulleys to bring the knots down onto the cuff. The two free suture limbs are then used to produce suture bridges over the tendon, using a Pushlock (Arthrex, Naples, FL), placed 1 cm distal to the lateral edge of the footprint (Fig 11, 12).

**Figure 13:**
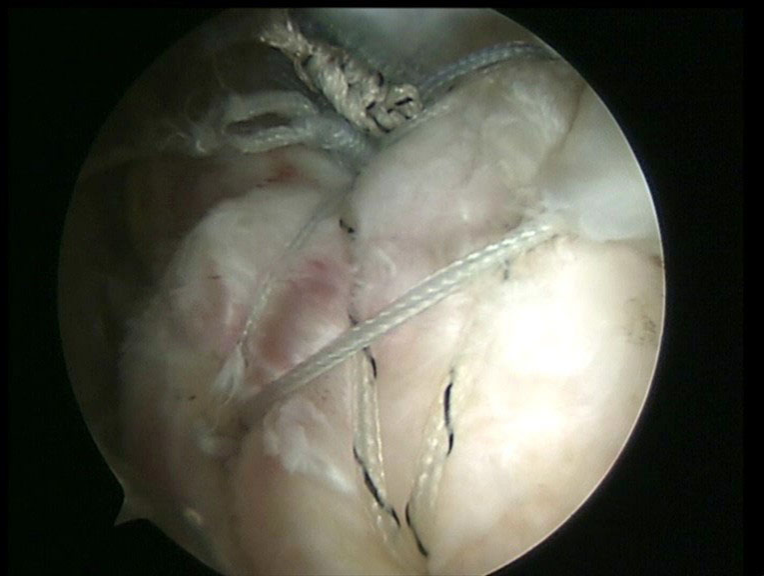
The final results.

The same double pulley – suture bridges technique is repeated for the other two suture limbs from the two medial anchors (Fig 13).

### Post-operative management

The arm is supported in a sling with an abduction pillow for 6 weeks. Active elbow flexion and extension are allowed, but terminal extension is restricted. Passive external rotation is started from the first day after surgery, and maintained within a comfortable range. Overhead stretching is restricted until 6 weeks post-operatively to avoid damaging the repair. At six weeks, the sling is removed, and overhead stretching with a rope and pulley are started. Isokinetic strengthening and rehabilitation of the rotator cuff, deltoid and scapular stabilizers are initiated at 10 or 12 weeks after the operation. Rehabilitation is continued for 6 months. Heavy manual work and overhead activities are allowed after a good restoration of shoulder strength, which normally occurs 6 to 10 months after surgery.

## Discussion

We presented an arthroscopic soft tissue LHBT tenodesis associated with a Roman Bridge repair. This technique allows to perform a double pulley – suture bridges repair for a rotator cuff tear, associated with a soft tissue tenodesis for the management of LHBT pathology. It has the advantage to avoid the use of additional fixation to tenodese the LHBT, and it is simple and fast. Indeed, the tenodesis of the LHBT is performed just with the passage of a shuttle inside the LHBT, after passing it through the anterior portion of the rotator cuff. Suture fixation is needed to prevent distal tendon migration.

The LHBT is an unique tendon, being partially intra-articular [16-18]. The biceps brachii is a supinator of the forearm and a flexor of the elbow joint), but its function at the shoulder is still debated [16,17,19-21]. As a gliding tendon, the LHBT pulls as a mechanical belt around the humeral head, allowing it to move on the fixed tendon during motion at the shoulder joint [17,19]. The association of rotator cuff tears and biceps tendon lesions is frequent [3], and the tendon of the LHBT is a major source of shoulder pain [3].

Arthroscopic release of the LHBT is a reliable management option for patients with chronic, recalcitrant biceps tendinopathy [12], and an alternative to tenodesis. It is not recommended in physical labourers, young individuals, and athletes because of the 38% incidence of symptoms of fatigue and discomfort, and loss of strength [12]. Tenotomy may not be the ideal intervention for patients of all ages with various shoulder abnormalities, but it appears acceptable for a specifically selected individuals, particularly in light of the decreased incidence of biceps tenderness when compared to tenodesis, which has unacceptable outcome in 6%–40% of patients [19]. It is a quick procedure that does not require additional fixation. Disadvantages associated with LHBT tenotomy can be distal migration of the LHBT with cosmetic deformity (Popeye sign) and impaired shoulder strength [12]. It has been suggested that loss of strength is related more to discomfort and those patients that have minimal pain, do not demonstrate a loss of strength. Tenotomy has not been shown to develop muscular atrophy, probably due to the intact short head [12].

On the other hand, tenodesis of the LHBT is suggested in young, active patients with a partial lesion of the LHBT, medial subluxation of the biceps tendon, and unreparable SLAP lesion [2,3,11]. Biceps tenodesis has potential advantages over tenotomy. These include prevention of muscle atrophy, maintenance of the length-tension relationship, maintenance of elbow flexion and supination strength, avoidance of cramping pain and avoidance of cosmetic deformity [2,3,11]. Arthroscopic tenodesis of the LHBT can be performed positioning a bio-interference screw into a bone socket [8,11,22]. A soft tissue arthroscopic tenodesis can be performed incorporating the LHBT into the rotator cuff repair, avoiding the use of a separate fixation technique for the biceps [12,13].

All these tenodesis techniques require the detachment of the biceps from the glenoid tubercle.

There have been studies on strength of tendon attachment with various tenodesis techniques. The available literature on this topic does not allow definitive conclusion, as no level I evidence has been produced to compare soft tissue tenodesis of the LHBT and tenodesis of the LHBT performed with additional fixation. Also, there are not level I studies to ascertain whether tenodesis of the LHBT performed with additional fixation is clinically superior to soft tissue tenodesis of the LHBT. There is a belief that additional tendon to bone fixation provides superior strength than soft tissue attachment initially, but this may not apply after a period of healing.

We associated a soft tissue tenodesis in the suture of a Roman Bridge repair for rotator cuff tear [15]. The Roman Bridge "double pulley-suture bridges" repair maximizes the good point of two techniques. In addition to the strong medial fixation obtained using a double pulley [3,23-25], the sutures bridges improve compression contact area and mean footprint pressure, provide a barrier to the synovial fluid from the joint to the healing area of tendon and bone, and share the load between fixation points [26]. By providing suture bridges of fixation, the number of points of fixation is increased, increasing the strength of the initial repair construct, and decreasing the load which each suture loop and knot must resist and the stress at each suture-cuff contact point [26-29]. The Roman Bridge "double pulley-suture bridges" repair may provide greater potential for osseous incorporation and healing at the tendon-bone interface by increasing the repair site area and thus greater ultimate strength of the repair compared with a single row suture anchor repair [15,30].

This is the first reported technique of soft tissue tenodesis of the LHBT associated with suture bridges repair for rotator cuff tear. The main advantage of our technique of soft tissue tenodesis of the LHBT is that it is performed with a simple passage of a shuttle through the biceps, with successive detachment of the LHBT from the glenoid. It is a technically easy procedure which can be performed relatively quickly, and does not require additional fixation.

## Conclusion

Additional biomechanical and clinical investigations are needed. Nevertheless, this technique of soft tissue tenodesis of the LHBT associated with suture bridges repair for rotator cuff tear is a viable option for the arthroscopic management of associated LHBT and rotator cuff tears.

## Competing interests

The authors declare that they have no competing interests.

## Authors' contributions

FF, UGL, NM and VD conceived the study. UGL, LR, and GR performed the review of the literature and wrote the initial draft. They also consented the patients whose photos are shown in this manuscript. FF, NM and VD advised on the practicalities of the surgery. All authors read and approved the final manuscript.

## Pre-publication history

The pre-publication history for this paper can be accessed here:


